# Screening for and Managing the Person with Frailty in Primary Care: ICFSR Consensus Guidelines

**DOI:** 10.1007/s12603-020-1492-3

**Published:** 2020-10-17

**Authors:** J. G. Ruiz, E. Dent, John E. Morley, R. A. Merchant, J. Beilby, J. Beard, C. Tripathy, M. Sorin, S. Andrieu, I. Aprahamian, H. Arai, M. Aubertin-Leheudre, J. M. Bauer, M. Cesari, L.-K. Chen, A. J. Cruz-Jentoft, P. De Souto Barreto, B. Dong, L. Ferrucci, R. Fielding, L. Flicker, J. Lundy, J. Y. Reginster, L. Rodriguez-Mañas, Y. Rolland, A. M. Sanford, A. J. Sinclair, J. Viña, D. L. Waters, C. Won Won, J. Woo, B. Vellas

**Affiliations:** 1 Geriatric Medicine, University of Miami Miller School of Medicine, Miami, Florida USA; 2 Adelaide and Baker Heart and Diabetes Institute, Torrens University of Australia, Melbourne, Australia; 3 Division of Geriatric Medicine, Saint Louis University School of Medicine, St. Louis, Missouri USA; 4 Division Geriatric Medicine, Department of Medicine, National University Hospital, National University Health System, Singapore, Singapore; 5 Think Education, Torrens University, Adelaide, Australia; 6 Centre of Excellence in Population Ageing Research, University of New South Wales, Sydney, Australia; 7 Primary Care Center and Division of General Medicine, University of Texas Health San Antonio, San Antonio, Texas USA; 8 University of Florida College of Medicine, Gainesville, Florida USA; 9 Department of Epidemiology, UMR1027 Inserm — Toulouse University III, University Toulouse, Toulouse, France; 10 Division of Geriatric Medicine, Jundiai Medical School, Department of Internal Medicine, Sao Paulo, Brazil; 11 Department of Psychiatry, University of Groningen, University Medical Center Groningen, Groningen, The Netherlands; 12 National Center for Geriatrics and Gerontology, Obu, Japan; 13 Dept des Sciences de l’activité physique, CRIUGM, Université du Québec à Montréal, Montréal, QC Canada; 14 Center for Geriatric Medicine and Network Aging Research, Heidelberg University; Agaplesion Bethanien Krankenhaus, Heidelberg, Germany; 15 IRCCS Istituti Clinici Scientifici Maugeri, University of Milan, Milano, Italy; 16 Center for Geriatrics and Gerontology, Taipei Veterans General Hospital, Taipei, Taiwan and Aging and Health Research Center, National Yang Ming University, Taipei, Taiwan; 17 Servicio de Geriatria, Hospital Universitario (IRYCIS), Ramón y Cajal, Madrid, Spain; 18 Gérontopôle of Toulouse, Institute of Ageing, Toulouse University Hospital (CHU Toulouse) and UPS/Inserm UMR 1027, University of Toulouse, Toulouse, Toulouse, France; 19 National Clinical Research Center for Geriatrics, West China Hospitals of Sichuan University, Chengdu, China; 20 National Institute on Aging/NTH, Baltimore, Maryland 21224 USA; 21 Jean Mayer USDA, Human Nutrition Research Center on Aging at Tufts University, Exercise Physiology, and Claude C. Pepper Older Americans Independence Center, Boston, Massachusetts USA; 22 Western Australia Centre for Health and Ageing Medical School, University of Western Australia, Perth, Australia; 23 Perry County Memorial Hospital, Perryville, Missouri USA; 24 Department of Public Health, Epidemiology and Health Economics, and Chair for Biomarkers of Chronic Diseases, Biochemistry Department of College of Sciences, King Saud University Riyadh, KSA, University of Liege, Liege, Belgium; 25 Hospital Universitario de Getafe, Servicio de Geriatria, Madrid, Spain; 26 Service de Médecine Interne et Gérontologie, Gerontopole, Toulouse, France; 27 Foundation for Diabetes Research in Older People, and Visiting Chair in Diabetes Care, King’s College, London, United Kingdom; 28 Freshage Research Group Leader, Dept Physiology Faculty Medicine, University of Valencia, Valencia, Spain; 29 Department of Medicine and School of Physiology, University of Otago, Dunedin, New Zealand; 30 Elderly Frailty Research Center, Department of Family Medicine, College of Medicine, Kyung Hee University, Seoul, South Korea; 31 Department of Medicine, the Chinese University of Hong Kong, Hong Kong, China; 32 Division of Geriatric Medicine, Saint Louis University, SLUCare Academic Pavilion, Section 2500 1008 S. Spring Ave., 2nd Floor, St. Louis, MO 63110 USA

**Keywords:** Frailty, primary care, aging

## Introduction

Frailty is now a well-recognized and common syndrome among older persons (1–3). Frailty is a syndrome which increases the risk of an older person to develop disability or to die when exposed either to physical or psychosocial stressors (4, 5). Although frailty, disability and multimorbidity often coexist and interact, they are distinct and separate concepts (6). Growing evidence suggests that each of these interrelated conditions is preventable and their associated complications manageable (6–8). However, early identification is imperative as once disability and multimorbidity occur, frailty in less likely to be prevented or reversed (9–11). As such it should be distinguished from persons with disability in their activities of daily living. The conditions leading to the frailty syndrome should have some degree of reversibility, thus distinguishing it from multimorbidity (7, 8, 12). Recently, the International Conference of Frailty and Sarcopenia Research (ICFSR) formulated evidence-based guidelines for the identification and management of physical frailty (13). Physical frailty was originally defined and validated by Fried et al (12, 14). This definition included measurements of low activity level, slowness of walking, muscle weakness, exhaustion and weight loss. This approach differs from that of Rockwood and Mitnitski (15) which used the number of “deficits” (signs, symptoms, clinical conditions) to determine a frailty index. Primary care represents the entry point into the health care system for many older adults who may be pre-frail and frail. A shortage of geriatricians and the higher frequency of frailty in community settings call for primary care clinicians (general practitioners, generalists, family physicians) to increasingly assess and manage older adults at risk for frailty or who are already frail.

The purpose of this paper is to suggest practical frailty screening and management strategies in primary care settings. We will also discuss the characteristics of these instruments and their applicability to primary care. For the sake of consistency hereafter, we will refer to clinicians delivering primary care as primary care providers.

## Screening (Case Finding

Primary care providers around the world report high patient workloads. The average primary care physician spends between less than a minute on consultations in Bangladesh to over 20 minutes in Sweden (16). Less than half of these physicians spend more than 10 minutes for consultations. The short amount of time physicians spent with older persons makes it extremely difficult to identify and develop a comprehensive diagnostic and management plan for geriatric syndromes. Primary care providers need easy and rapid approaches to help them identify patients with frailty. Below we describe time-efficient and validated screening tools that clinicians can use to identify frailty in older persons in primary care.

**Figure 1:**
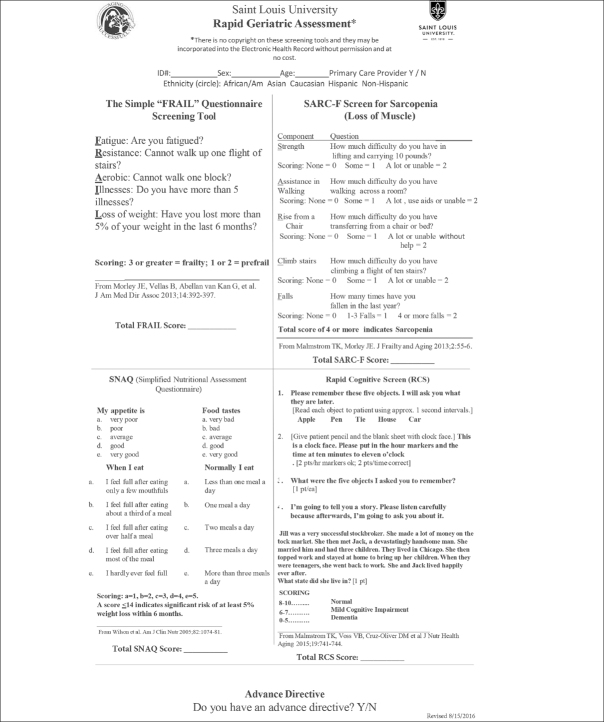
The FRAIL and Other Components of the Rapid Geriatric Assessment

The FRAIL scale (Figure 1) is a simple 5-item questionnaire that can be answered in 15 to 30 seconds (17, 18). In persons over 50 years of age the FRAIL Scale predicted disability and mortality at 9 years (19, 20). It performed as well as the Fried Frailty Phenotype (16a) and the Study of Osteoporosis Fractures (SOF). In the Australian Longitudinal Study on Women’s Health, the FRAIL scale predicted future disability over a 15-year period in middle aged women (21). A large study in Hong Kong demonstrated that FRAIL predicted over 4 years both disability and mortality as well as the CHS scale and the Rockwood Frailty Index (22). FRAIL predicted mortality in the Survey of Health Aging and Retirement in Europe (SHARE)(23). Numerous other studies have validated the predictive capacity of FRAIL (24–28). Thus, the FRAIL scale is now recommended as a screen tool for older persons visiting primary care providers in Australia (29) and in Brazil (30). An adapted version of the tool has also been developed for nursing homes (i.e., FRAIL-NH), which has shown to be predictive of adverse outcomes in the long term care setting (31, 32).

Another rapid screening test for frailty is the Clinical Frailty Scale (CFS) (33–35) . The CFS scale consists of 9 items and is available in a pictorial version with corresponding text. It is correlated with the Frailty Index and is predictive of mortality (33, 36). The first three items refer to persons that are non-frail, item four assesses vulnerability whereas items five to eight include an assessment of disability. It is uncertain how correctly the average clinician can classify persons in the different categories (especially distinguishing frail from the disabled) by using the Clinical Frailty Scale (CFS) and without falling into the risk of subjectivity.

The Vulnerable Elders Survey-13 (VES-13) consists of questions to recognize older persons with frailty (37, 38). The VES-13 questionnaire consists of items measuring activities of daily living, physical function, self-rated health, and one question on age. It is a practical and brief screening tool that can be staff-administered or self-administered in less than 5 minutes. It has been demonstrated to be a good predictor of decreased function and death in older persons (39, 40).

The Kihon checklist was introduced by the Japanese long-term care insurance system in 2006 as an evaluation of frailty (41, 42). It consists of 25 yes or no questions that evaluate the domains of physical function, nutrition, feeding, social activity, memory, mood and lifestyle (43). It has been validated against the Fried frailty phenotype (41). The Kihon checklist is predictive of mortality (44) and shows good diagnostic accuracy in identifying frailty in primary care based on a recent Australian study (45).

The VES-13 (37, 38) and Kihon checklist (41, 42) include items assessing basic and instrumental activities of daily living among their scoring items. As with the Clinical Frailty Scale, clinicians using these instruments may have difficulties at distinguishing frailty from disability.

The World Health Organization (WHO) has focused on developing an approach to screen persons for decreases in intrinsic capacity, defined as “the combination of the individual’s physical and mental, including psychological, capacities” (46). To screen for loss of intrinsic capacity they have developed the “Integrated Care for Older People” (ICOPE) instrument (47, 48). Primary care providers should match for frailty development due to physical inactivity during the COVID-19 pandemic (47). While not specifically designed to identify frailty and having no designated cutoff to distinguish frailty states, the screening test can be delivered by a professional screener or by patient self-assessment using either a mobile application (App) or the BOTFRAIL (an internet conversational robot). The ICOPE screening test consists of 6 areas including measurements of cognition, mobility, malnutrition, vision impairment, hearing loss and depression. (Table 1)

**Table 1:** Screening Tool for the “Integrated Care for Older Persons” (ICOPE)

**Priority Conditions Associated with Declines in Intrinsic Capacity**	**Tests**
Cognitive Decline	1. Remember three words: Flower, door, rice (for example) 2. Orientation in time and space: What is the full date today? Where are you now (home, clinic, etc.)? 3. Recalls the three words?
Limited Mobility	Chair rise test: Rise from chair five times without using arms. Did the person complete five chair rises within 14 seconds?
Malnutrition	1. Weight loss: Have you unintentionally lost more than 3 kg over the last three months? 2. Appetite loss: Have you experienced loss of appetite?
Visual Impairment	Do you have any problems with your eyes: Difficulties in seeing far, reading, eye diseases or currently under medical treatment (e.g., diabetes, high blood pressure)?
Hearing Loss	Hears whispers (whisper test) or Screening audiometry result is 35 dB or less or Passes automated app-based digits-in-noise test
Depressive Symptoms	Over the past two weeks, have you been bothered by • Feeling down, depressed or hopeless? • Little interest or pleasure in doing things?

The Study of Osteoporotic Fractures (SOF) frailty scale was developed and validated in an all-female cohort. It consists of three items that are easy to administer: the ability to rise from an armless chair five times (inability = 1); response to the question “Do you feel full of energy?” (answer of “no” = 1); and weight loss > 5% in the past year (presence of weight loss = 1). Each item is scored as 0 for normal or 1 for abnormal (Prefrail =1, and Frail = 2 or 3) (49).» The SOF can be easily incorporated into a primary care practice and is useful in the identification of patients who may require referral for comprehensive geriatric assessment.

Frailty indexes that are automatically generated from electronic health records or administrative claims data may offer distinct advantages to busy primary care providers. As electronic health records become increasingly ubiquitous in primary care practices in high income countries, clinicians can use this information at the point of care to identify patients with frailty. Recently developed electronic frailty indexes have demonstrated predictive validity for hospitalizations, nursing home placement, cost of care, prediction and resource allocation to care for populations in value-based care delivery (50–53). A limitation is that electronic health records may not yet be widely available in many low- and middle-income countries. Furthermore, they might rely on medical data of limited relevance for the older person, and ignore aspects of critical importance in geriatric patients (e.g., functional status).

## Referral to Comprehensive Geriatric Assessment (CGA)

Investigators have often validated frailty screening instruments against the CGA (54). Screening instruments serve to identify those older adults who may be at risk for frailty or may have already developed frailty. Although many frailty screening instruments are sensitive, these tools often display low specificity (55). Thus, screening tests require confirmation of frailty with more thorough evaluations of the older person such as those part of a CGA. Geriatric assessment may uncover previously unrecognized problems that may contribute to the development or progression of frailty in older adults (56–58). Timely identification of these problems may lead clinicians to design and implement personalized interventions which can improve patient outcomes (57, 58). At the same time, it is important to remind that the CGA is a process diagnostically and therapeutically. The assessments conducted in the first part of the CGA to identify the persons critical aspects should always be followed by a multi-disciplinary and integrated intervention to make the methodology meaningful.

## Management of Frailty

There is a growing evidence in support of a variety of interventions that target older adults with frailty in primary care settings. Research indicates that exercise, nutrition and geriatric assessment represent effective, evidence-based interventions in primary care. A recent meta-analysis of 31 studies including 4794 participants concluded that resistance exercise, with or without nutrition supplementation may improve the frailty status of older adults in primary care settings. In older subjects with diabetes and frailty, resistance exercise as part of a multimodal approach significantly improved physical performance over one year measured by the short performance physical battery (SPPB) which was accompanied by a significant decrease in healthcare expenditure (59).

Comprehensive Geriatric Assessment was also more effective than control groups at reducing frailty (58). Older adults with frailty often display prolonged periods of sedentary behaviors (60). Interventions to reduce overall sedentary behavior in older people with frailty may include short bouts of physical activity after intervals of uninterrupted inactivity (13, 31). Although less studied, other clinical interventions such as nutrition may offer benefits to older adults with frailty in outpatient settings. Observational studies suggest potential benefits of the Mediterranean diet (61, 62) and of vitamin D supplementation in patients that are deficient (63, 64). A summary of these recommendations can be seen in Table 2.

**Table 2:** Management of Frailty in Primary Care

**Primary Prevention**	**Secondary Prevention**	**Tertiary Prevention**
1. Provide community education including television, newspapers, magazines and social media to do aerobic and resistance exercise regularly 2. Health care professionals to regularly reinforce the importance of exercise. 3. Community lectures by health care professionals on the importance of exercise 4. Yearly screening with a rapid screen for frailty (FRAIL or ICOPE)	If positive frailty screen: 1. Check for and treat possible reversible causes as in Table 1 or 2 2. Enroll in an exercise program 3. Advise on adequate (leucine enriched) protein intake 4. Consider grip strength, 4m gait speed and short physical performance battery	1. Check ADLs and IADLs 2. Refer for comprehensive geriatric assessment 3. Refer to physical and occupational therapy 4. Optimize home environment 5. Provide a long term exercise program

The following sections give an overview of two examples of management approaches implemented in primary care settings.

The Rapid Geriatric Assessment: A management program for the different components of the FRAIL has been developed at Saint Louis Lniversity and is being developed into an App (20, 22). For fatigue, common causes are depression, sleep apnea, hypotension, anemia, hypothyroidism, hypoxia and vitamin B12 deficiency (65). Persons who have trouble completing the resistance and aerobic questions can be referred to multicomponent exercise program for sarcopenia (49, 66). They may also benefit from a leucine enriched essential amino acid supplement (67). Persons with more than five illnesses should have their medications reviewed to see if they are on inappropriate medications for older persons or if they have polypharmacy, where reduction of some medicines may improve their function (68–71). Older persons with weight loss should be examined for treatable causes of weight loss as delineated by the MEALS-ON-WHEELS mnemonic (8). In addition, the use of a caloric supplement can be considered (72) (Table 3). The FRAIL screen has been integrated with 3 other tests: The SARC-F (Sarcopenia) (2, 73), the Simplified Nutrition Appetite Questionnaire (SNAQ) (74, 75) and the Rapid Cognitive Screen (RCS) (76) to provide a more comprehensive geriatric examination, which can be performed by a primary care provider or other allied health care professionals (Figure 1). The complete RGA can be carried out in under 5 minutes (77) and is available as an App which was utilized by the National Lniversity Health System in Singapore (78). Furthermore, the RGA can be integrated into the Medicare Annual Wellness Visit (79).

**Table 3:** Diagnostic and Management Program for an Older Individual who has Deficits on the FRAIL Questionnaire (Copyright Saint Louis Lniversity and John E. Morley)

**Potential Deficits**
Fatigue: Exclude Depression
Exclude Sleep Apnea
Measure TSH, Vitamin B^12^ and Hemoglobin
Exclude low blood pressure or orthostasis resistance or Aerobic: Aerobic and Resistance exercise
Leucine enriched essential amino acid supplement
Measure bioavailable vitamin D and replace if low
Illnesses: Remove inappropriate medications including those causing side effects
Reduce Polypharmacy
Loss of Weight: Exclude depression
Stop drugs causing weight loss
Check for elderly abuse
Is the person paranoic (late life paranoia) or afraid being overweight will kill them?
Does the person have dysphagia?
Are there oral problems making chewing difficult?
Does the person have a nosocomial infection, e.g., *Helicobacter pylori* or tuberculosis?
Does the person have dementia?
Does the person have hyperthyroidism, Addison’s disease or pheochromocytoma?
Does the person have celiac disease or pancreatic insufficiency?
Does the person have eating difficulties?
Is person on low salt, low cholesterol or other therapeutic diet?
Does the person have cholecystitis?

The Integrated Care for Older People: The ICOPE program may be indicated for older persons that are either pre-frail or frail. The ICOPE rationale to target older persons at the pre-frail stage is that early interventions aimed at reversing pre-frailty or preventing the patient from becoming frail are more likely to be successful. It may also reduce the need to implement a higher number of step by step approaches which may be more suited to older persons who are already frail. In the ICOPE program, the older person is referred to either a primary care provider or a trained nurse to complete a geriatric assessment that includes a personalized intervention plan which is reassessed every 4 months. The follow up reassessments can be performed remotely through telemedicine (80). Each time the team detects a worsening of one or more ICOPE functions, they proceed to evaluate the reasons for the deficit (step 2 ICOPE) and propose personalized interventions (step 3). The ICOPE program encompasses medical, environmental and social domains. Moreover, older persons participation and empowerment are integral parts of the ICOPE program. Older persons learn how to self-assess their ICOPE functions using self-managements tools, apps or conversational bots (automated computer programs that interact with humans) (80). Digital medicine, e-health and telemedicine technologies offer healthcare teams efficient ways to monitor ICOPE functions and intervene in a timely fashion when indicated. For example, as part of the INSPIRE program a nurse monitors older persons’ functional status by reviewing databases every 4 months. If new abnormalities are detected, the nurse refers the older person to the primary care provider for step 2. The primary care provider can then choose to implement step 2 part during a routine clinical encounter, ask a trained nurse to perform a more comprehensive geriatric assessment, or contact a geriatrician for a tele-expertise consultation. The primary care provider uses the results of the cognitive and frailty scales to decide whether it is appropriate to refer the patient to a geriatrician (48, 80, 81). Another possible approach is to utilize the Korean Frailty Index for primary Care (82).

## Frailty within a Primary Care Model of Care

The optimal management of an older person with frailty in primary care requires a coordinated and integrated approach. Primary care providers need to work in collaboration with multidisciplinary teams which involve geriatricians, allied health professionals (including physiotherapists, dieticians, exercise physiologists, social workers, and occupational therapists), caregivers and the patient themselves. A model of care that is widely adopted in the US is the patient-centered medical home model (PCMH) (83). The principles that guide this model are relevant to the care of older adults with frailty ensuring the delivery of comprehensive care, that is patient-centered, coordinated, accessible, safe and of high quality (84, 85). The PCMH model may provide an organizing framework for the implementation of screening and management strategies by primary care providers (86, 87). Within this model, primary care providers lead a team of professionals to ensure comprehensive and coordinated care for older adults with frailty.

## The Role of Education and Training

Key to the success of frailty screening and management initiatives in primary care is participation of competent and motivated primary care providers (88). Education and training of the workforce represent crucial approaches to increase the uptake of screening and management for frailty in primary care (89). Success of these initiatives will demand that undergraduate, graduate and continuing professional development training programs for medical and allied health practitioners include these topics in their curricula.

## Conclusion

A number of rapid screening tests have been developed to evaluate frailty in the older population. These tests are predictive of poor clinical outcomes. Screening and managing frailty appear to be reasonable approaches to reducing disability in older persons. It is important to adapt our health care system to the aging of the population and move from the traditional disease-oriented medical model to a more global and modern patient-centered model that encompasses the assessment, monitoring and maintenance of function with the ultimate goal of preventing frailty and disability.

## References

[1] Morley JE, Vellas B, van Kan GA, Anker SD, Bauer JM, Bernabei R (2013). Frailty consensus: a call to action. J Am Med Dir Assoc..

[2] Dent E, Morley JE, Cruz-Jentoft AJ, Arai H, Kritchevsky SB, Guralnik J (2018). International Clinical Practice Guidelines for Sarcopenia (ICFSR): Screening, Diagnosis and Management. J Nutr Health Aging..

[3] Walston J, Bandeen-Roche K, Buta B, Bergman H, Gill TM, Morley JE (2019). Moving Frailty Toward Clinical Practice: NIA Intramural Frailty Science Symposium Summary. J Am Geriatr Soc..

[4] Dent E, Martin FC, Bergman H, Woo J, Romero-Ortuno R, Walston JD (2019). Management of frailty: opportunities, challenges, and future directions. Lancet.

[5] Morley JE (2011). Frailty: diagnosis and management. J Nutr Health Aging..

[6] Fried LP, Ferrucci L, Darer J, Williamson JD, Anderson G (2004). Untangling the concepts of disability, frailty, and comorbidity: implications for improved targeting and care. J Gerontol A Biol Sci Med Sci..

[7] Ferrucci L, Giallauria F, Schlessinger D (2008). Mapping the road to resilience: novel math for the study of frailty. Mech Ageing Dev..

[8] Morley JE (2017). The New Geriatric Giants. Clin Geriatr Med..

[9] Ferrucci L, Guralnik JM, Studenski S, Fried LP, Cutler GB, Walston JD (2004). Designing randomized, controlled trials aimed at preventing or delaying functional decline and disability in frail, older persons: a consensus report. J Am Geriatr Soc..

[10] Espinoza SE, Jung I, Hazuda H (2012). Frailty transitions in the San Antonio Longitudinal Study of Aging. J Am Geriatr Soc..

[11] Pollack LR, Litwack-Harrison S, Cawthon PM, Ensrud K, Lane NE, Barrett-Connor E (2017). Patterns and Predictors of Frailty Transitions in Older Men: The Osteoporotic Fractures in Men Study. J Am Geriatr Soc..

[12] Fried LP, Tangen CM, Walston J, Newman AB, Hirsch C, Gottdiener J (2001). Frailty in older adults: evidence for a phenotype. J Gerontol A Biol Sci Med Sci..

[13] Dent E, Morley JE, Cruz-Jentoft AJ, Woodhouse L, Rodriguez-Manas L, Fried LP (2019). Physical Frailty: ICFSR International Clinical Practice Guidelines for Identification and Management. J Nutr Health Aging..

[14] Newman AB, Gottdiener JS, McBurnie MA, Hirsch CH, Kop WJ, Tracy R (2001). Associations of subclinical cardiovascular disease with frailty. J Gerontol A Biol Sci Med Sci..

[15] Rockwood K, Mitnitski A (2007). Frailty in relation to the accumulation of deficits. J Gerontol A Biol Sci Med Sci..

[16] Irving G, Neves AL, Dambha-Miller H, Oishi A, Tagashira H, Verho A (2017). International variations in primary care physician consultation time: a systematic review of 67 countries. BMJ Open..

[17] Abellan van Kan G, Rolland Y, Bergman H, Morley JE, Kritchevsky SB, Vellas B (2008). The I.A.N.A Task Force on frailty assessment of older people in clinical practice. J Nutr Health Aging..

[18] Abellan van Kan G, Rolland YM, Morley JE, Vellas B (2008). Frailty: toward a clinical definition. J Am Med Dir Assoc..

[19] Malmstrom TK, Miller DK, Morley JE (2014). A comparison of four frailty models. J Am Geriatr Soc..

[20] Morley JE, Malmstrom TK, Miller DK (2012). A simple frailty questionnaire (FRAIL) predicts outcomes in middle aged African Americans. J Nutr Health Aging..

[21] Susanto M, Hubbard RE, Gardiner PA (2018). Validity and Responsiveness of the FRAIL Scale in Middle-Aged Women. J Am Med Dir Assoc..

[22] Woo J, Leung J, Morley JE (2012). Comparison of frailty indicators based on clinical phenotype and the multiple deficit approach in predicting mortality and physical limitation. J Am Geriatr Soc..

[23] Theou O, Brothers TD, Pena FG, Mitnitski A, Rockwood K (2014). Identifying common characteristics of frailty across seven scales. J Am Geriatr Soc..

[24] Hyde Z, Flicker L, Almeida OP, Hankey GJ, McCaul KA, Chubb SA (2010). Low free testosterone predicts frailty in older men: the health in men study. J Clin Endocrinol Metab..

[25] Ravindrarajah R, Lee DM, Pye SR, Gielen E, Boonen S, Vanderschueren D (2013). The ability of three different models of frailty to predict all-cause mortality: results from the European Male Aging Study (EMAS). Arch Gerontol Geriatr..

[26] Woo J, Yu R, Wong M, Yeung F, Wong M, Lum C (2015). Frailty Screening in the Community Using the FRAIL Scale. J Am Med Dir Assoc..

[27] Chode S, Malmstrom TK, Miller DK, Morley JE (2016). Frailty, Diabetes, and Mortality in Middle-Aged African Americans. J Nutr Health Aging..

[28] Salminen M, Viljanen A, Horanta S, Viikari P, Wuorela M, Vahlberg T, et al. Frailty and mortality: an 18-year follow-up study among Finnish community-dwelling older people. Aging Clin Exp Res. 2019. 10.1007/s40520-019-01383-4 PMC7532963 31654244

[29] Frailty detection — ‘a game changer’ for older Australians Australia2018 [Available from: https://www.agedcareguide.com.au/talking-aged-care/frailty-detection-a-game-changer-for-older-australians.

[30] Lourenço RA, Moreira VG, Mello R, Santos I, Lin SM, Pinto ALF (2018). Consenso brasileiro de fragilidade em idosos: conceitos, epidemiologia e instrumentos de avaliação. Geriatrics, Gerontology and Aging..

[31] Kehler DS, Clara I, Hiebert B, Stammers AN, Hay JL, Schultz A (2018). The association between bouts of moderate to vigorous physical activity and patterns of sedentary behavior with frailty. Exp Gerontol..

[32] Kaehr EW, Pape LC, Malmstrom TK, Morley JE (2016). FRAIL-NH Predicts Outcomes in Long Term Care. J Nutr Health Aging..

[33] Ritt M, Schwarz C, Kronawitter V, Delinic A, Bollheimer LC, Gassmann KG (2015). Analysis of Rockwood et Al’s Clinical Frailty Scale and Fried et Al’s Frailty Phenotype as Predictors of Mortality and Other Clinical Outcomes in Older Patients Who Were Admitted to a Geriatric Ward. J Nutr Health Aging..

[34] Rockwood K, Song X, MacKnight C, Bergman H, Hogan DB, McDowell I (2005). A global clinical measure of fitness and frailty in elderly people. CMAJ.

[35] Theou O, van der Valk AM, Godin J, Andrew MK, McElhaney JE, McNeil SA, et al. Exploring clinically meaningful changes for the frailty index in a longitudinal cohort of hospitalized older patients. J Gerontol A Biol Sci Med Sci. 2020. 10.1093/gerona/glaa084 PMC7518565 32274501

[36] Basic D, Shanley C (2015). Frailty in an older inpatient population: using the clinical frailty scale to predict patient outcomes. J Aging Health..

[37] Belmin J, Khellaf L, Pariel S, Jarzebowski W, Valembois L, Zeisel J (2020). Validation of the French version of the Vulnerable Elders Survey-13 (VES-13). BMC Med Res Methodol..

[38] Bongue B, Buisson A, Dupre C, Beland F, Gonthier R, Crawford-Achour E (2017). Predictive performance of four frailty screening tools in community-dwelling elderly. BMC Geriatr..

[39] Arora VM, Johnson M, Olson J, Podrazik PM, Levine S, Dubeau CE (2007). Using assessing care of vulnerable elders quality indicators to measure quality of hospital care for vulnerable elders. J Am Geriatr Soc..

[40] Min LC, Elliott MN, Wenger NS, Saliba D (2006). Higher vulnerable elders survey scores predict death and functional decline in vulnerable older people. J Am Geriatr Soc..

[41] Yamada Y, Nanri H, Watanabe Y, Yoshida T, Yokoyama K, Itoi A (2017). Prevalence of Frailty Assessed by Fried and Kihon Checklist Indexes in a Prospective Cohort Study: Design and Demographics of the Kyoto-Kameoka Longitudinal Study. J Am Med Dir Assoc..

[42] Kunimoto M, Shimada K, Yokoyama M, Matsubara T, Aikawa T, Ouchi S (2019). Relationship between the Kihon Checklist and the clinical parameters in patients who participated in cardiac rehabilitation. Geriatr Gerontol Int..

[43] Nemoto M, Yabushita N, Kim MJ, Matsuo T, Seino S, Tanaka K (2012). Assessment of vulnerable older adults’ physical function according to the Japanese Long-Term Care Insurance (LTCI) system and Fried’s criteria for frailty syndrome. Arch Gerontol Geriatr..

[44] Kojima G, Taniguchi Y, Kitamura A, Shinkai S (2018). Are the Kihon Checklist and the Kaigo-Yobo Checklist Compatible With the Frailty Index?. J Am Med Dir Assoc..

[45] Ambagtsheer RC, Visvanathan R, Dent E, Yu S, Schultz TJ, Beilby J (2020). Commonly Used Screening Instruments to Identify Frailty Among Community-Dwelling Older People in a General Practice (Primary Care) Setting: A Study of Diagnostic Test Accuracy. J Gerontol A Biol Sci Med Sci..

[46] Cesari M, Araujo de Carvalho I, Amuthavalli Thiyagarajan J, Cooper C, Martin FC, Regjnster JY (2018). Evidence for the Domains Supporting the Construct of Intrinsic Capacity. J Gerontol A Biol Sci Med Sci..

[47] Sanchez-Rodriguez D, Annweiler C, Gillain S, Vellas B. Implementation of the Integrated Care of Older People (ICOPE) App in Primary Care: New Technologies in Geriatric Care During Quarantine of COVID-19 and Beyond. The Journal of Frailty &amp; Aging. 2020:1. 10.14283/jfa.2020.24 PMC7195618 33575702

[48] Takeda C, Guyonnet S, Sumi Y, Vellas B, Araujo de Carvalho I (2020). Integrated Care for Older People and the Implementation in the INSPIRE Care Cohort. J Prev Alzheimers Dis..

[49] Ensrud KE, Ewing SK, Taylor BC, Fink HA, Cawthon PM, Stone KL (2008). Comparison of 2 frailty indexes for prediction of falls, disability, fractures, and death in older women. Arch Intern Med..

[50] Clegg A, Bates C, Young J, Ryan R, Nichols L, Ann Teale E (2016). Development and validation of an electronic frailty index using routine primary care electronic health record data. Age Ageing..

[51] Orkaby AR, Nussbaum L, Ho YL, Gagnon D, Quach L, Ward R (2019). The Burden of Frailty Among U.S. Veterans and Its Association With Mortality, 2002–2012. J Gerontol A Biol Sci Med Sci..

[52] Johnston KJ, Wen H, Joynt Maddox KE (2020). Relationship of a Claims-Based Frailty Index to Annualized Medicare Costs: A Cohort Study. Ann Intern Med..

[53] Kim DH, Patorno E, Pawar A, Lee H, Schneeweiss S, Glynn RJ (2020). Measuring Frailty in Administrative Claims Data: Comparative Performance of Four Claims-Based Frailty Measures in the U.S. Medicare Data. J Gerontol A Biol Sci Med Sci..

[54] Pialoux T, Goyard J, Lesourd B (2012). Screening tools for frailty in primary health care: a systematic review. Geriatr Gerontol Int..

[55] Clegg A, Rogers L, Young J (2015). Diagnostic test accuracy of simple instruments for identifying frailty in community-dwelling older people: a systematic review. Age Ageing..

[56] Morley JE, Adams EV (2015). Rapid Geriatric Assessment. J Am Med Dir Assoc..

[57] Pilotto A, Custodero C, Maggi S, Polidori MC, Veronese N, Ferrucci L (2020). A multidimensional approach to frailty in older people. Ageing Res Rev..

[58] Macdonald SH, Travers J, She EN, Bailey J, Romero-Ortuno R, Keyes M (2020). Primary care interventions to address physical frailty among community-dwelling adults aged 60 years or older: A meta-analysis. PLoS One..

[59] Rodriguez-Manas L, Laosa O, Vellas B, Paolisso G, Topinkova E, Oliva-Moreno J (2019). Effectiveness of a multimodal intervention in functionally impaired older people with type 2 diabetes mellitus. J Cachexia Sarcopenia Muscle..

[60] Blodgett J, Theou O, Kirkland S, Andreou P, Rockwood K (2015). The association between sedentary behaviour, moderate-vigorous physical activity and frailty in NHANES cohorts. Maturitas..

[61] Feng Z, Lugtenberg M, Franse C, Fang X, Hu S, Jin C (2017). Risk factors and protective factors associated with incident or increase of frailty among community-dwelling older adults: A systematic review of longitudinal studies. PLoS One..

[62] Wang Y, Hao Q, Su L, liu Y, Liu S, Dong B (2018). Adherence to the Mediterranean Diet and the Risk of Frailty in Old People: A Systematic Review and Meta-Analysis. J Nutr Health Aging..

[63] Beaudart C, Buckinx F, Rabenda V, Gillain S, Cavalier E, Slomian J (2014). The effects of vitamin D on skeletal muscle strength, muscle mass, and muscle power: a systematic review and meta-analysis of randomized controlled trials. J Clin Endocrinol Metab..

[64] Buchebner D, Bartosch P, Malmgren L, McGuigan FE, Gerdhem P, Akesson KE (2019). Association Between Vitamin D, Frailty, and Progression of Frailty in Community-Dwelling Older Women. J Clin Endocrinol Metab..

[65] Morley JE, von Haehling S, Anker SD, Vellas B (2014). From sarcopenia to frailty: a road less traveled. J Cachexia Sarcopenia Muscle..

[66] Bauer J, Morley JE, Schols A, Ferrucci L, Cruz-Jentoft AJ, Dent E (2019). Sarcopenia: A Time for Action. An SCWD Position Paper. J Cachexia Sarcopenia Muscle..

[67] Kim CO, Lee KR (2013). Preventive effect of protein-energy supplementation on the functional decline of frail older adults with low socioeconomic status: a community-based randomized controlled study. J Gerontol A Biol Sci Med Sci..

[68] Tjia J, Velten SJ, Parsons C, Valluri S, Briesacher BA (2013). Studies to reduce unnecessary medication use in frail older adults: a systematic review. Drugs Aging..

[69] Rolland Y, Morley JE (2016). Editorial: Frailty and Polypharmacy. J Nutr Health Aging..

[70] Potter K, Flicker L, Page A, Etherton-Beer C (2016). Deprescribing in Frail Older People: A Randomised Controlled Trial. PLoS One..

[71] Khezrian M, McNeil CJ, Murray AD, Myint PK (2020). An overview of prevalence, determinants and health outcomes of polypharmacy. Ther Adv Drug Saf..

[72] Warne C, Forrester IT, Jones L, Morley JE E (2019). Screening for the Anorexia of Aging. J Nutr Health Aging..

[73] Bahat G, Oren MM, Yilmaz O, Kilic C, Aydin K, Karan MA (2018). Comparing SARC-F with SARC-CalF to Screen Sarcopenia in Community Living Older Adults. J Nutr Health Aging..

[74] Ilhan B, Bahat G, Oren MM, C BK, Durmazoglu S, Karan MA (2018). Reliability and validity of Turkish version of the Simplified Nutritional Appetite Questionnaire (SNAQ). J Nutr Health Aging..

[75] Mohammadi MR, Akhondzadeh S, Keshavarz SA, Mostafavi SA (2019). The Characteristics, Reliability and Validity of the Persian Version of Simplified Nutritional Appetite Questionnaire (SNAQ). J Nutr Health Aging..

[76] Malmstrom TK, Voss VB, Cruz-Oliver DM, Cummings-Vaughn LA, Tumosa N, Grossberg GT (2015). The Rapid Cognitive Screen (RCS): A Point-of-Care Screening for Dementia and Mild Cognitive Impairment. J Nutr Health Aging..

[77] Sanford AM, Morley JE, Berg-Weger M, Lundy J, Little MO, Leonard K (2020). High prevalence of geriatric syndromes in older adults. PLoS One..

[78] Merchant RA, Hui RJY, Kwek SC, Sundram M, Tay A, Jayasundram J (2020). Rapid Geriatric Assessment Using Mobile App in Primary Care: Prevalence of Geriatric Syndromes and Review of Its Feasibility. Front Med (Lausanne).

[79] Morley JE, Abele P (2016). The Medicare Annual Wellness Visit in Nursing Homes. J Am Med Dir Assoc..

[80] Tavassoli N, Piau A, Berbon C, De Kerimel J, Lafont C, Barreto PDS, et al. Framework Implementation Of The Inspire ICOPE-Care Program In Collaboration With The World Health Organization (Who) In The Occitania Region. 2020. 10.14283/jfa.2020.26 33575698

[81] de Souto Barreto P, Guyonnet S, Ader I, Andrieu S, Casteilla L, Davezac N, et al. The INSPIRE research initiative: A program for geroscience and healthy aging research going from animal models to humans and the healthcare system. The Journal of Frailty & Aging. 2020:1–8. 10.14283/jfa.2020.18 33575696

[82] Won CW, Lee Y, Lee S, Kim M (2020). Development of Korean Frailty Index for Primary (KFI-PC) and its criterion validity. Annals of Geriatric Med and Res.

[83] Grumbach K, Grundy P (2010). Outcomes of implementing patient centered medical home interventions.

[84] Ferrante JM, Balasubramanian BA, Hudson SV, Crabtree BF (2010). Principles of the patient-centered medical home and preventive services delivery. Ann Fam Med..

[85] Rich E, Lipson D, Libersky J, Parchman M (2012). Coordinating care for adults with complex care needs in the patient-centered medical home: Challenges and solutions.

[86] Stange KC, Nutting PA, Miller WL, Jaén CR, Crabtree BF, Flocke SA (2010). Defining and measuring the patient-centered medical home. J Gen Intern Med..

[87] Rich EC, Lipson D, Libersky J, Peikes DN, Parchman ML (2012). Organizing care for complex patients in the patient-centered medical home. Ann Fam Med..

[88] Cesari M, Prince M, Thiyagarajan JA, De Carvalho IA, Bernabei R, Chan P (2016). Frailty: An Emerging Public Health Priority. J Am Med Dir Assoc..

[89] Windhaber T, Koula ML, Ntzani E, Velivasi A, Rizos E, Doumas MT (2018). Educational strategies to train health care professionals across the education continuum on the process of frailty prevention and frailty management: a systematic review. Aging Clin Exp Res..

